# Dataset of chronic intracranial EEG of epilepsy patients via responsive neurostimulation system

**DOI:** 10.3389/fnins.2026.1815732

**Published:** 2026-05-08

**Authors:** Chen Feng, Haoqi Ni, Zhoule Zhu, Hongjie Jiang, Zhe Zheng, Wenjie Ming, Shuang Wang, Kedi Xu, Junming Zhu

**Affiliations:** 1 Department of Neurosurgery, The Second Affiliated Hospital Zhejiang University School of Medicine, Hangzhou, China; 2 College of Biomedical Engineering and Instrument Science, Zhejiang University, Hangzhou, China; 3 Epilepsy Center, The Second Affiliated Hospital Zhejiang University School of Medicine, Hangzhou, China; 4 Zhejiang Key Laboratory of Research and Transformation for Major Neurosurgical Diseases, Hangzhou, China; 5 Clinical Brain-Computer Interface Zhejiang Provincial Engineering Research Center, Hangzhou, China; 6 Department of Neurology, The Second Affiliated Hospital Zhejiang University School of Medicine, Hangzhou, China; 7 State Key Laboratory of Brain-machine Intelligence, Zhejiang University, Hangzhou, China

**Keywords:** dataset, epilepsy, intracranial electroencephalography, neuromodulation, responsive neurostimulation

## 1 Introduction

Epilepsy is a chronic neurological disorder affecting over 70 million people globally (Thijs et al., 2019), with approximately one-third developing drug-resistant epilepsy (DRE) (Kwan et al., 2011; Kwan and Sander, 2004; Chen et al., 2018). For these patients, neuromodulation has emerged as a critical alternative (Ryvlin et al., 2014; Kwon et al., 2018). The responsive neurostimulation (RNS) system is a closed-loop brain–computer interface that continuously monitors intracranial EEG (iEEG) and delivers automated electrical stimulation upon detecting epileptiform activity (Morrell, 2011; Wong et al., 2019). Clinical trials demonstrate sustained efficacy, with a median 75% seizure reduction, a 73% responder rate, and over one-third of patients achieving ≥90% reduction over nine years (Nair et al., 2020; Roa et al., 2023; Bergey et al., 2015).

Beyond therapy, RNS devices generate longitudinal “stimulate–response” iEEG data during chronic treatment, providing a unique platform for studying epilepsy network dynamics (Boddeti et al., 2022). Studies leveraging RNS recordings have revealed multidien cycles in seizure likelihood (Khambhati et al., 2024; Baud et al., 2018; Leguia et al., 2021) and frequency-specific network reorganization tied to treatment response (Khambhati et al., 2021). Despite these advances, publicly available chronic stimulus-linked iEEG datasets remain scarce (Rao, 2021). Existing repositories, including iEEG.org (Wagenaar et al., 2015), the Temple University Hospital EEG Corpus (Shah et al., 2018), and the SWEC-ETHZ dataset (Burrello et al., 2019), do not provide stimulate–response recordings from closed-loop neuromodulation devices.

Here we present a chronic iEEG dataset from eight DRE patients implanted with RNS systems. Each recording spans 90 s of dual-channel iEEG covering pre-stimulation (~60 s), stimulation, and post-stimulation (~30 s) intervals, with stimulation parameter annotations and electrode metadata. All data are organized in iEEG-BIDS format (Holdgraf et al., 2019; Gorgolewski et al., 2016) and released on OpenNeuro (Feng et al., 2025; Markiewicz et al., 2021). The dataset offers: (1) long-term iEEG from chronically implanted RNS devices, extending beyond short-term presurgical monitoring; (2) structured stimulate–response data with precise temporal alignment between stimulation events and iEEG; and (3) clinically adjusted stimulation parameters across sessions, reflecting individualized therapeutic adaptations.

## 2 Material and methods

### 2.1 Patients

This dataset comprises iEEG data collected from eight patients with drug-resistant focal epilepsy (four males, four females; mean age: 38 ± 10.6). Detailed patient clinical characteristics—including epilepsy subtype, disease duration, seizure onset zone (SOZ) location, surgical history and stimulation targets—are provided in Supplementary Table S1. The therapeutic response after RNS system implant of all eight patients is provided in Supplementary Table S4. All patients were hospitalized at the Second Affiliated Hospital of Zhejiang University School of Medicine (SAHZU) to undergo implantation of the RNS systems. All patients gave their consent to share their de-identified data publicly and signed the informed consent form. The study was approved by the Chinese Clinical Trial Registry (ChiCTR2200055247) and the Ethics Committee of the Second Affiliated Hospital of Zhejiang University School of Medicine (20210226).

### 2.2 Pre-surgical evaluation and implantation

**Figure 1:**
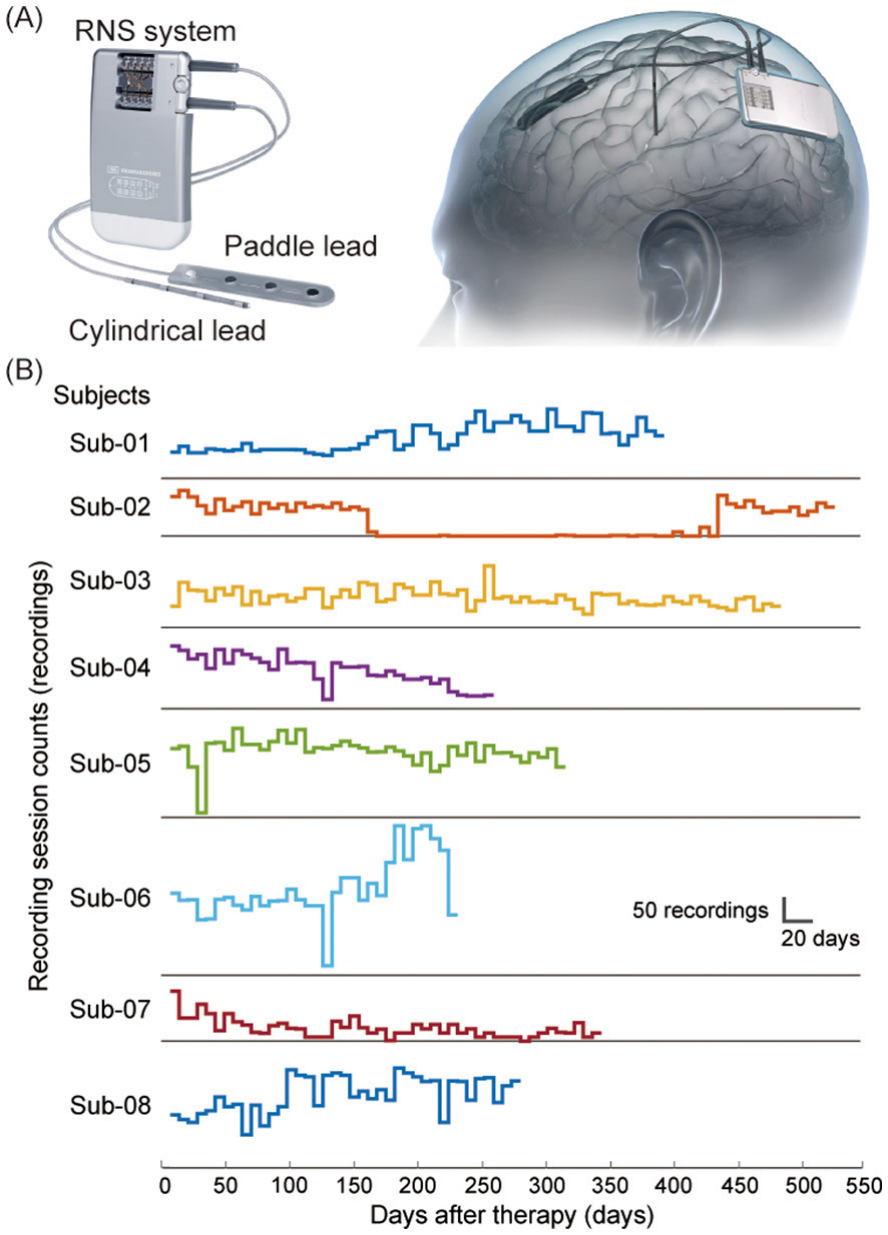
Dataset information overview. **(A)** RNS system and implantation. **(B)** Recording session counts across patients.

Prior to RNS implantation, all patients underwent temporary intracranial electrode placement for several weeks of electrophysiological monitoring to identify the seizure onset zone. The SOZ was confirmed through multidisciplinary consultation involving experienced neurosurgeons and neurologists. These SOZ subsequently served as the surgical targets for the implantation of RNS stimulation leads. The eight patients were implanted with the Epilecure™ system (Figure 1, an RNS device manufactured by GenLight MedTech Co., Ltd., Hangzhou, China). During surgery, neurosurgeons implanted either the cylindrical leads (targeting deep brain structures; four contacts per lead, 10 mm inter-contact spacing, 2 mm exposed length; GenLight MedTech Co., Ltd., Hangzhou, China) or the paddle leads (targeting cerebral cortex structures; four contacts per lead, 10 mm inter-contact spacing, 2.9 mm exposed length; GenLight MedTech Co., Ltd., Hangzhou, China) at the identified epileptogenic foci in both hemispheres of the brain (Figure 1).

### 2.3 Electrodes localization

Electrode positions were determined by co-registering preoperative T1-weighted MRI with postoperative CT. Cortical surface reconstruction was performed using FreeSurfer (FIS, 1999), and electrode coordinates were verified in BrainStorm (Tadel et al., 2011).

### 2.4 Seizure onset detection and stimulation

Approximately 1 month (31.5 ± 9.2 days) post implantation surgery, the RNS system was activated for responsive seizure onset detection and therapeutic intervention. The system operated on a closed-loop mechanism: it continuously recorded iEEG signals via specific contacts on designated unilateral or bilateral channels and performed real-time seizure detection using a line-length algorithm. Specifically, the detector computed the cumulative absolute amplitude change (line length) of the iEEG signal within a sliding detection window; when the computed value exceeded a patient-specific pre-set threshold, the system identified the event as a potential seizure onset (acknowledging that RNS devices possess a notable false-positive rate and do not capture 100% of clinical seizures). Subsequently, the device delivered electrical stimulation with a pre-programmed fixed duration per episode. The key programmable stimulation parameters included current amplitude (mA), frequency (Hz), pulse width (μs), and pulse train duration (ms), all using charge-balanced biphasic waveforms. These parameters were individually configured for each patient. A summary of stimulation parameters across all eight patients is provided in Supplementary Table S3. Based on seizure activity observed over the preceding two weeks, detection thresholds and stimulation parameters were adjusted during regular clinical follow-ups by experienced neurosurgeons and neurologists. The categories of parameters modified during follow-ups are documented in the Supplementary material.

### 2.5 IEEG data recording

A timestamp was generated at the onset of each stimulation episode. The system recorded a total of 90 s of iEEG data for each event, comprising approximately 60 s before the onset of stimulation, the stimulation period itself, and the remaining post-stimulation interval (~20 s of physiologically interpretable signal). Stimulation artifact duration ranged from approximately 1–5 s, scaling with the programmed stimulation duration. We recommend that users adopt a conservative post-stimulation analysis window starting ≥5 s after stimulation onset; users should consult the *events.tsv* files to identify artifact-free segments, particularly for recordings containing multiple stimulation events. Following a defined refractory period, this cycle resumed to intervene in subsequent supra-threshold events. All iEEG signals were digitized at a sampling rate of 200 Hz and processed using a hardware-determined band-pass filter of 1.5–159 Hz.

### 2.6 IEEG data selection

Inclusion criteria required: (1) continuous tracking exceeding 6 months, a threshold that captures parameter optimization and meaningful seizure reduction (Bergey et al., 2015; Nair et al., 2020) as well as long-term network reorganization (Khambhati et al., 2021); and (2) high signal quality. For each session, we selected the single day with the highest seizure-event count (mean: 16.95 events) and widest temporal dispersion (time span between earliest and latest detection events), with coverage of both daytime (06:00–18:00) and nighttime (18:00–06:00) verified. All recordings from the selected day were included.

## 3 Dataset overview

All raw data were converted into an iEEG dataset named RNS_Epilepsy-iBIDS in iEEG-BIDS format (Holdgraf et al., 2019; Gorgolewski et al., 2016) and released on OpenNeuro (Feng et al., 2025; Markiewicz et al., 2021). All personally identifiable information has been removed and subject order has been randomized. The dataset structure is shown in Supplementary Figure S1. The dataset contains 6,019 iEEG recordings from 355 sessions of eight patients, with a total recording duration of 154.6 h and a size of 497.76 MB. Raw data in European Data Format (.edf) were converted to BIDS using the *data2bids* function from FieldTrip (Oostenveld et al., 2011). The dataset was validated using the BIDS Validator (https://github.com/bids-standard/bids-validator).

### 3.1 Patient and session folders

Each patient's directory contains multiple session folders, labeled with numerical suffixes that reflect their chronological order. The number of sessions varies across patients and depends on the total duration of longitudinal monitoring. Within each session folder, an iEEG subdirectory is included, which stores all iEEG recordings acquired on the selected day of that corresponding week.

### 3.2 IEEG folder

IEEG recordings are stored within a session-specific directory for each patient (e.g., sub-01/ses-01). This directory contains all iEEG–related data and metadata, organized as follows.

#### 3.2.1 iEEG recording files

For each seizure-related recording (files labeled **_*task-seizure*_run-**, with the run number indicating chronological order), the primary iEEG signals are provided in European Data Format (*ieeg.edf* ). Each recording is accompanied by a sidecar JSON file (*ieeg.json*), describing modality-specific metadata, including electrical stimulation parameters (amplitude, frequency, pulse width, and duration), manufacturer and institution information, and acquisition settings.

#### 3.2.2 Channel information

A channel-level description file (*channels.tsv*) is provided for every seizure recording. This file lists all recording contacts using a standardized naming convention (e.g., EEG001,1+,3-), specifies electrode type (DBS or ECoG), documents anatomical implantation sites, and includes acquisition parameters such as units, sampling frequency, and filter cutoffs (high-pass and low-pass).

#### 3.2.3 Electrical stimulation event annotations

Electrical stimulation events for each seizure recording are documented in *events.tsv*, which includes the onset, duration, and offset of each stimulation epoch. The first stimulation marker typically appears near 60 s, corresponding to a stimulation artifact in the signal. However, a single 90-s iEEG segment may contain more than one stimulation event. When the RNS device detects two seizure events in close temporal proximity, multiple stimulations may be delivered within a short interval. Therefore, additional stimulation markers may occur either before or after the 60-s point.

#### 3.2.4 Electrode localization data

Clinical electrode coordinates and dimensions are provided in *electrodes.tsv*, along with a corresponding *coordsystem.json* file describing the coordinate system. All eight patients in this dataset were localized using the ACPC coordinate system with measurements in millimeters. Electrode names indicate both the hemisphere and the site of implantation.

## 4 Data validation

### 4.1 Data quality validation

Technical validation was performed in MATLAB (R2024b, The MathWorks, Natick, Massachusetts, USA). All quantitative checks used the 10 s of iEEG preceding stimulation onset in each 90-s recording. Figure 2 summarizes results for sub-01; the same pipeline was applied to all patients (Supplementary Figures S5–S11).

- **Visual inspection**. Two independent reviewers (C.F., H.N.) screened all channels and sessions for non-physiological artifacts. This step aimed to identify and exclude non-stimulation-related noise and artifacts, thereby ensuring high signal quality.
- **Longitudinal band-limited power**. Absolute power in Delta (1–4 Hz), Theta (4–8 Hz), Alpha (9–12 Hz), Beta (13–30 Hz), and Gamma (31–80 Hz) was computed per event, averaged within each session, and plotted vs. session index (both linear and log scale) to track slow drifts over chronic follow-up.
- **Inter-session PSD similarity**. Session-level PSDs (Welch's method; 0.64 s Hamming window, 78.1% overlap) were compared via pairwise Pearson correlations, yielding a session-by-session correlation matrix and adjacent-session spectral-stability indices.

**Figure 2:**
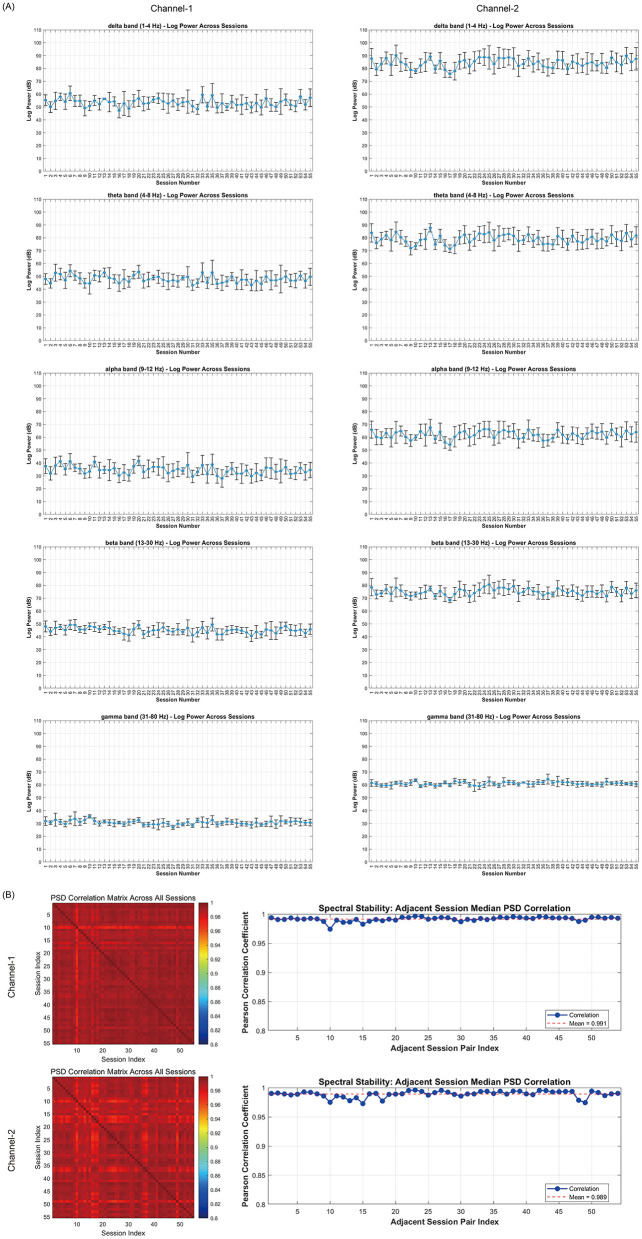
Data quality validation for participant sub-01 (example). **(A)** Longitudinal band power: absolute power in Delta (1–4 Hz), Theta (4–8 Hz), Alpha (9–12 Hz), Beta (13–30 Hz), and Gamma (31–80 Hz) from the 10-s pre-stimulation window, averaged within each session, plotted vs. session index; log power across sessions for all bands. **(B)** PSD similarity: pairwise correlation matrix of session PSDs; spectral stability as Pearson correlation between adjacent sessions' median PSDs. The same pipeline was applied to all patients (Supplementary Figures S5–S11).

### 4.2 Scientific utility demonstration

To demonstrate the dataset's research utility, we performed pre- vs. post-stimulation comparisons at two longitudinal stages: therapy onset and ~6 months of chronic RNS. Frequency bands were Delta (1–4 Hz), Theta (4–8 Hz), Alpha (9–12 Hz), Beta (13–30 Hz), and Gamma (31–80 Hz). PSDs were computed with Welch's method (MATLAB pwelch; 0.64 s Hamming window, 78.1% overlap). Where noted, paired *t*-tests compared pre- vs. post-stimulation metrics; Bonferroni correction was applied across the five frequency bands.

- **Power spectral analysis (Supplementary Figure S2)**. Using sub-02 as an example, we compared ses-01 (beginning of RNS) and ses-30 (~6 months) for both iEEG channels, with stimulation aligned at *t* = 0 s on a ±15 s epoch. Pre- and post-stimulation analysis windows were 10 s segments (−15 to −5 s and +5 to +15 s), avoiding the stimulation artifact. At ses-01, both channels exhibited a prominent β-band peak (~20 Hz) with similar pre- and post-stimulation spectra. After 6 months, that β prominence diminished while pre/post spectral shapes within ses-30 remained aligned, consistent with longitudinal network reorganization under chronic RNS (Khambhati et al., 2021).
- **Phase locking value (Supplementary Figure S3)**. Using sub-07 as an example (ses-01 vs. ses-26, ~6 months), phase locking value (PLV) (Aydore et al., 2013) quantified inter-channel phase synchrony. Dual-channel signals were band-pass filtered (Butterworth, zero-phase), instantaneous phases were obtained via the Hilbert transform, and phase differences were summarized in polar histograms **(A)**. **(B)** compares mean ± SD PLV across recordings before vs. after stimulation at each longitudinal stage. The most prominent pre/post change at 6 months was in the **Theta** band (*p* = 0.0115, uncorrected), consistent with modulation of pathological inter-regional synchrony (Piper et al., 2022).
- **Envelope length (Supplementary Figure S4)**. Using the same participant (sub-07, ses-01 vs. ses-26), envelope length (Munia and Aviyente, 2019; Esteller et al., 2001)—defined as $EL=\overset{N-1}{\underset{i=1}{\sum }}|A\left(i+1\right)-A\left(i\right)|$ on Hilbert envelopes *A*(*i*) (μV)—was computed for each frequency band. At therapy initiation, stimulation suppressed Gamma-band envelope length (*p* = 0.0112, uncorrected). After 6 months, envelope length in the Beta (*p* = 0.0461, uncorrected) and Gamma (*p* = 0.0201, uncorrected) bands increased post-stimulation, in line with acute desynchronization of epileptiform activity (Anderson et al., 2024). None of the individual comparisons survived Bonferroni correction for five frequency bands; these *p*-values are reported for descriptive purposes to illustrate the dataset's analytic potential.

## 5 Usage notes

### 5.1 Data notes

For patient sub-02, there is a notable absence of recorded events between the 7th and 14th months of treatment. This gap was attributed to a technical malfunction in the data storage module of the RNS device. The issue was subsequently rectified and did not compromise the delivery of therapeutic stimulation during this interval. Consequently, the final dataset includes a total of 37 sessions for this patient, a duration that remains consistent with our established inclusion criteria.

All iEEG data included in this dataset consist of events triggered by the RNS device under preset seizure detection algorithms. It is critical to note that these recordings do not universally represent clinical seizures. The frequency of actual clinical seizures is significantly lower than the volume of device-recorded events. Reactive closed-loop intracranial devices such as RNS, which must respond within seconds of seizure onset and cannot miss events, are normally tuned to have high sensitivity, which results in frequent false-positive stimulation (Baldassano et al., 2016, 2019).

### 5.2 Potential research applications

This dataset can support multiple research directions: (1) characterization of stimulation-evoked electrophysiological responses across different parameters; (2) biomarker discovery for treatment response prediction using longitudinal iEEG features; (3) development and benchmarking of seizure detection algorithms; and (4) optimization of adaptive closed-loop stimulation protocols.

### 5.3 Limitations

Users should be aware of the following limitations: (1) not all device-triggered events correspond to clinical seizures due to the false-positive rate of the detection algorithm; (2) the data selection strategy favored days with the highest seizure event frequency, which may not fully represent periods of low seizure activity; (3) the post-stimulation analysis window is limited to ~25 s of clean signal after accounting for stimulation artifacts; (4) each recording contains only two iEEG channels, limiting spatial resolution for network analyses.

## Data Availability

The datasets presented in this study can be found in online repositories. The names of the repository/repositories and accession number(s) can be found at: https://openneuro.org/datasets/ds007095.
