# Supplementary material for: Dataset of chronic intracranial EEG of epilepsy patients via responsive neurostimulation system
Source: Front Neurosci. 2026 May 8;20:1815732. doi: 10.3389/fnins.2026.1815732 (PMC13194392; doi:10.3389/fnins.2026.1815732)
Supplement: Supplementary file 1 [file Data_Sheet_1.pdf]

Supplementary Material

1 TABLES

Table S1. Participant clinical characteristics. TLE = temporal lobe epilepsy; M = male; F = female.

Subject	Age	Sex	Epilepsy subtype	Disease duration (years)	SOZ location	Prior surgical history	Stimulation target(s)
sub-01	39	M	TLE	19	Bilateral frontotemporal	None	Bilateral hippocampi
sub-02	55	F	TLE	33	Bilateral frontotemporal	None	Bilateral hippocampi
sub-03	33	F	TLE	24	Bilateral temporal	None	Bilateral hippocampi
sub-04	41	M	TLE	23	Bilateral frontotemporal	None	Bilateral hippocampi
sub-05	30	F	TLE	14	Bilateral temporal	None	Bilateral hippocampi
sub-06	54	M	TLE	29	Bilateral frontotemporal	None	Bilateral hippocampi
sub-07	30	M	TLE	20	Left posterior temporo-occipital, left fusiform gyrus	None	Left temporal lobe
sub-08	24	F	TLE	21	Bilateral hippocampi	None	Bilateral hippocampi

Table S2. Participant-level dataset summary statistics, including recording duration (days), number of sessions, total number of iEEG recordings, and total recording duration (hours) for all eight participants.

Subject	Record duration (days)	Number of sessions	Number of iEEG recordings	Total recording duration (hours)
sub-01	399	55	808	20.61
sub-02	531	37	426	10.84
sub-03	484	68	965	25.23
sub-04	288	35	556	14.25
sub-05	321	44	998	25.62
sub-06	315	31	886	22.71
sub-07	336	46	439	11.29
sub-08	286	39	941	24.04
Total	/	/	6019	154.60

Table S3. Electrical stimulation parameters for all subjects across sessions.

Subject	Sessions	Amplitude (mA)	Frequency (Hz)	No. of Sessions
Sub-01	01–55	2.5	200	55
Sub-02	01–37	2.0	200	37
Sub-03	1–34	4.5	200	34
	35–38	4.0	200	4
	39–68	3.0	5	30
Sub-04	1–9	2.5	200	9
	10–35	3.0	200	26
Sub-05	01–12	2.0	200	12
	13–21	3.0	200	9
	22–44	3.5	200	23
Sub-06	1–9	2.0	200	9
	10–21	3.0	200	12
	22–31	4.0	200	10
Sub-07	1–16	3.0	200	16
	17–39	4.0	200	23
	40–46	4.5	200	7
Sub-08	1–18	1.0	200	18
	19–33	1.5	200	15
	34–39	1.8	200	6

Table S4. Monthly seizure frequency after RNS implantation for all eight participants. Each participant has two rows: "Seizure" shows the monthly seizure count and "Session" shows the corresponding dataset session numbers. "/" indicates no data available. Baseline denotes the pre-RNS monthly seizure frequency. See Usage Notes in main text for sub-02 data gap.

		Baseline	Month-01	Month-02	Month-03	Month-04	Month-05	Month-06
Sub-01	Seizure	3.3	1	2	4	1	1	8
	Session	/	1–5	6–9	10–13	14–17	18–22	23–25
Sub-02	Seizure	6.3	18	6	9	12	3	2
	Session	/	1–5	6–9	10–13	14–17	18–22	23
Sub-03	Seizure	724.7	738	412	254	296	271	171
	Session	/	1–5	6–9	10–13	14–16	17–20	21–25
Sub-04	Seizure	4.0	4	3	0	5	4	1
	Session	/	1–4	5–8	9–12	13–16	17–20	21–24
Sub-05	Seizure	5.7	1	5	3	2	2	3
	Session	/	1–5	6–9	10–13	14–18	19–21	22–25
Sub-06	Seizure	6.7	16	10	10	11	8	10
	Session	/	1–3	4–7	8–10	11–14	15–17	18–22
Sub-07	Seizure	253.7	91	56	70	104	70	152
	Session	/	1–4	5–9	10–13	14–17	18–22	23–25
Sub-08	Seizure	201.0	60	51	54	45	33	39
	Session	/	1–5	6–8	9–12	13–17	18–21	22–25

Table S4. (*continued*)

		Baseline	Month-07	Month-08	Month-09	Month-10	Month-11	Month-12
Sub-01	Seizure	3.3	1	1	1	6	6	4
	Session	/	26–29	30–33	34–38	39–41	42–45	46–50
Sub-02	Seizure	6.3	/	/	/	/	/	/
	Session	/	/	/	/	/	/	/
Sub-03	Seizure	724.7	190	193	117	195	130	103
	Session	/	26–29	30–33	34–38	39–42	43–46	47–50
Sub-04	Seizure	4.0	0	0	4	0	/	/
	Session	/	25–27	28–30	31–33	34–35	/	/
Sub-05	Seizure	5.7	2	1	0	1	0	/
	Session	/	26–28	29–33	34–37	38–42	43–44	/
Sub-06	Seizure	6.7	13	/	10	12	13	/
	Session	/	23–24	/	25	26–30	31	/
Sub-07	Seizure	253.7	117	178	143	179	159	153
	Session	/	26–29	30–33	34–37	38–41	42–45	46
Sub-08	Seizure	201.0	34	37	27	20	/	/
	Session	/	26–29	30–33	34–38	39	/	/

		Baseline	Month-13	Month-14	Month-15	Month-16	Month-17	Month-18
Sub-01	Seizure	3.3	3	/	/	/	/	/
	Session	/	51–55	/	/	/	/	/
Sub-02	Seizure	6.3	/	/	0	0	0	0
	Session	/	/	/	24–26	27–30	31–34	35–37
Sub-03	Seizure	724.7	150	136	114	134	/	/
	Session	/	51–55	56–59	60–63	64–68	/	/

2 FIGURES

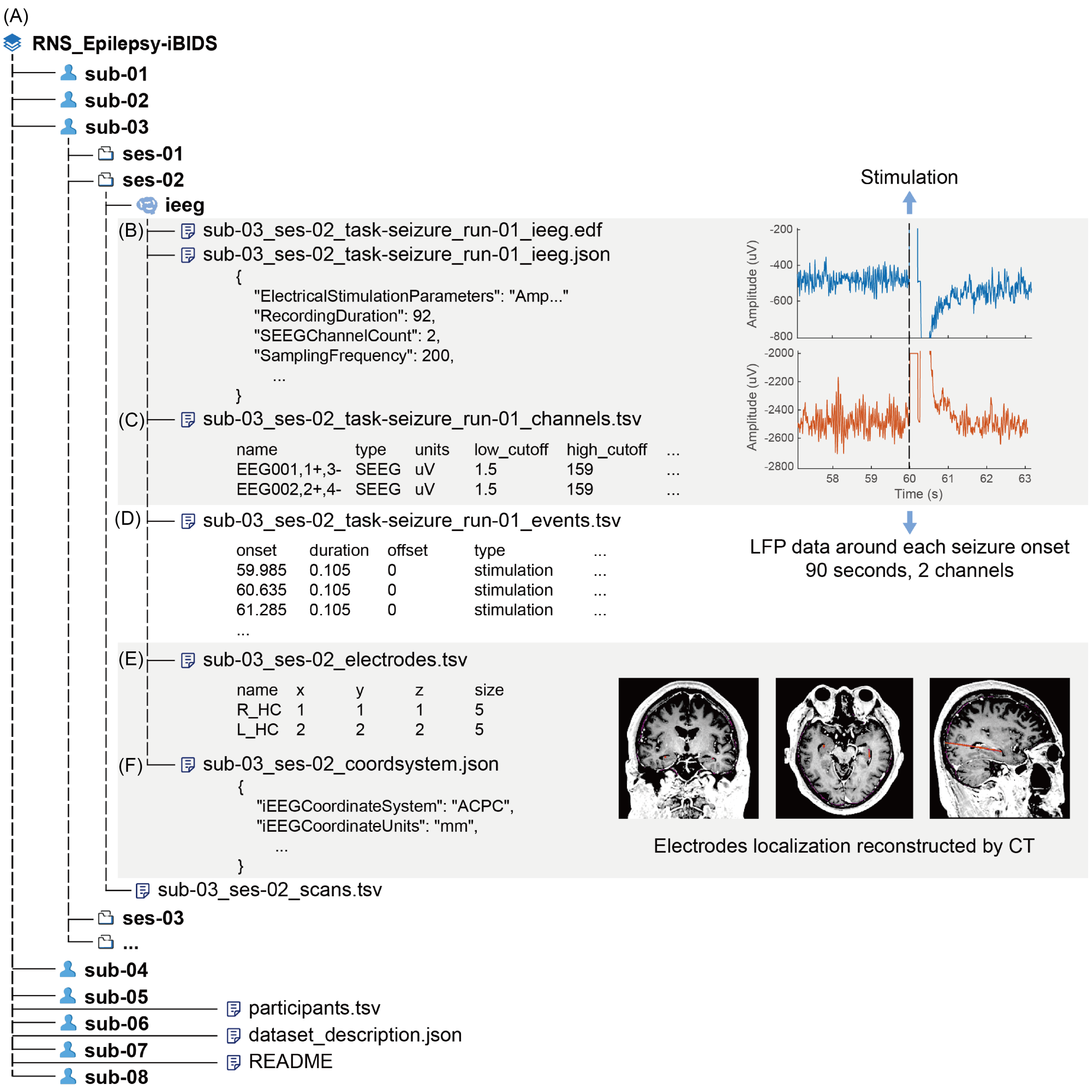

Figure S1: Structure of the dataset (RNS_Epilepsy-iBIDS) organized in iEEG-BIDS format. (**A**) Directory hierarchy: 8 participants (*sub-***), each containing multiple sessions (*ses-***). (**B**) iEEG recording files: dual-channel signals (*ieeg.edf*) and sidecar metadata (*ieeg.json*). (**C**) Channel description file (*channels.tsv*). (**D**) Electrical stimulation events (*events.tsv*). (**E**) Electrode localization and dimensions (*electrodes.tsv*). (**F**) Coordinate system description (*coordsystem.json*).

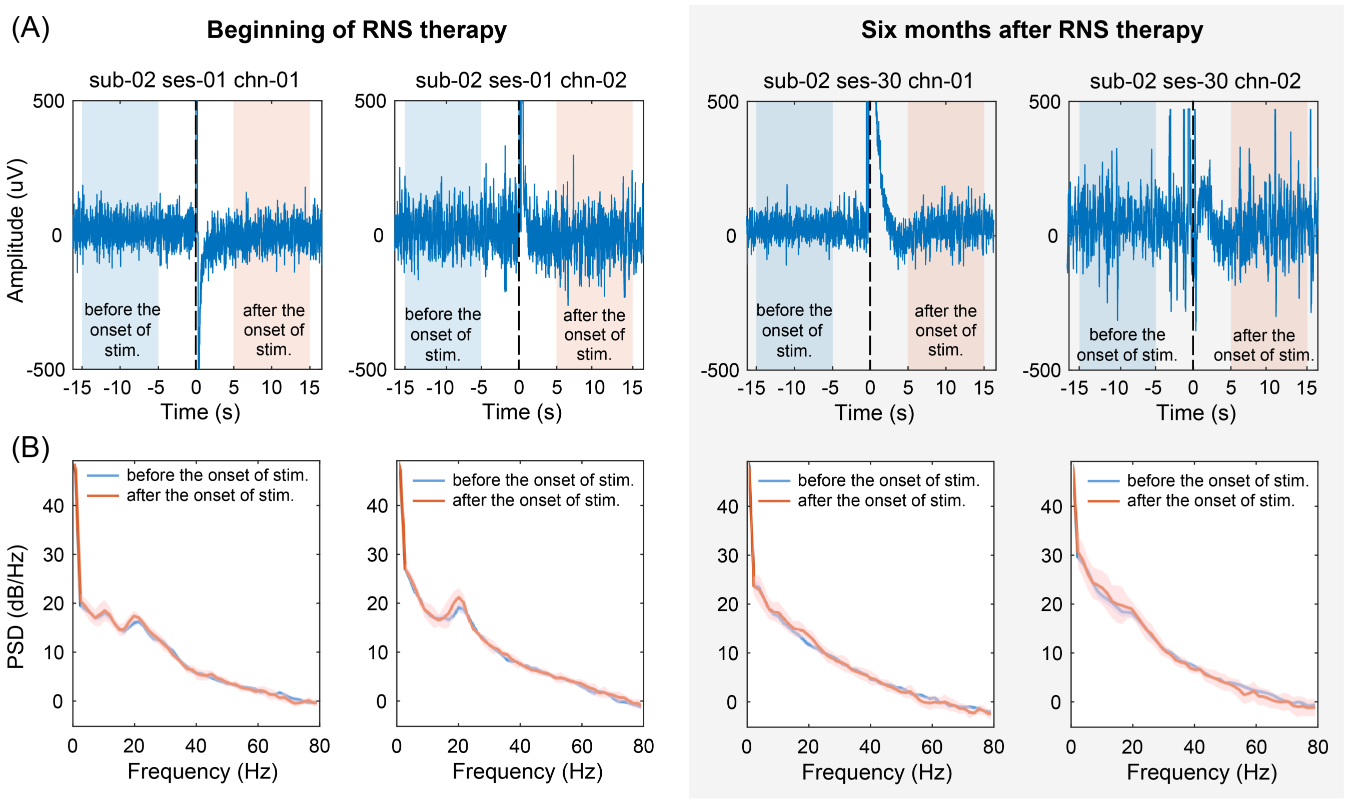

Figure S2: **Scientific utility (spectral):** participant sub-02. (**A**) Raw dual-channel iEEG traces (±15 s centered on stimulation at $t = 0$) for ses-01 (therapy onset, left) and ses-30 (∼six months, right). Blue- and orange-shaded background regions indicate the pre-stimulation (-15 to -5 s) and post-stimulation ($+5$ to $+15$ s) analysis windows, respectively. (**B**) Welch PSDs (0–80 Hz) computed within each analysis window (blue line: pre-stimulation; orange line: post-stimulation); shaded bands indicate variability across recordings within the session. At ses-01, both channels show a prominent β-band peak (∼20 Hz) with overlapping pre/post spectra; at ses-30 that peak is attenuated while pre/post spectral shapes within the session remain similar.

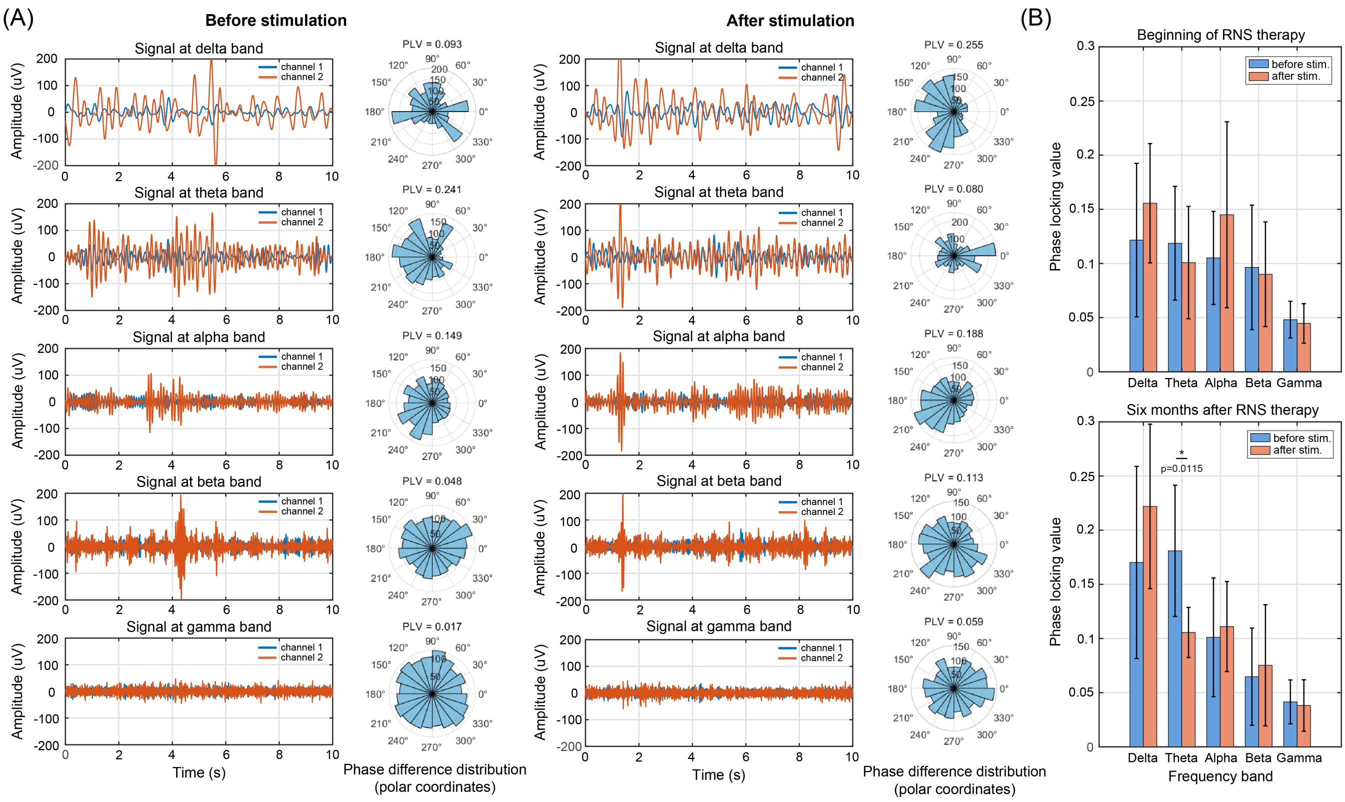

Figure S3: **Scientific utility (connectivity):** phase locking value (PLV) for participant sub-07 (ses-01 vs. ses-26, ∼six months). (**A**) For Delta through Gamma bands, filtered dual-channel iEEG traces (blue: channel 1; orange: channel 2) and polar histograms of inter-channel phase difference before versus after stimulation, with band-wise PLV values. (**B**) Mean ± SD PLV by frequency band at therapy initiation (ses-01, top) versus the six-month visit (ses-26, bottom), comparing windows before (blue) and after (orange) stimulation.

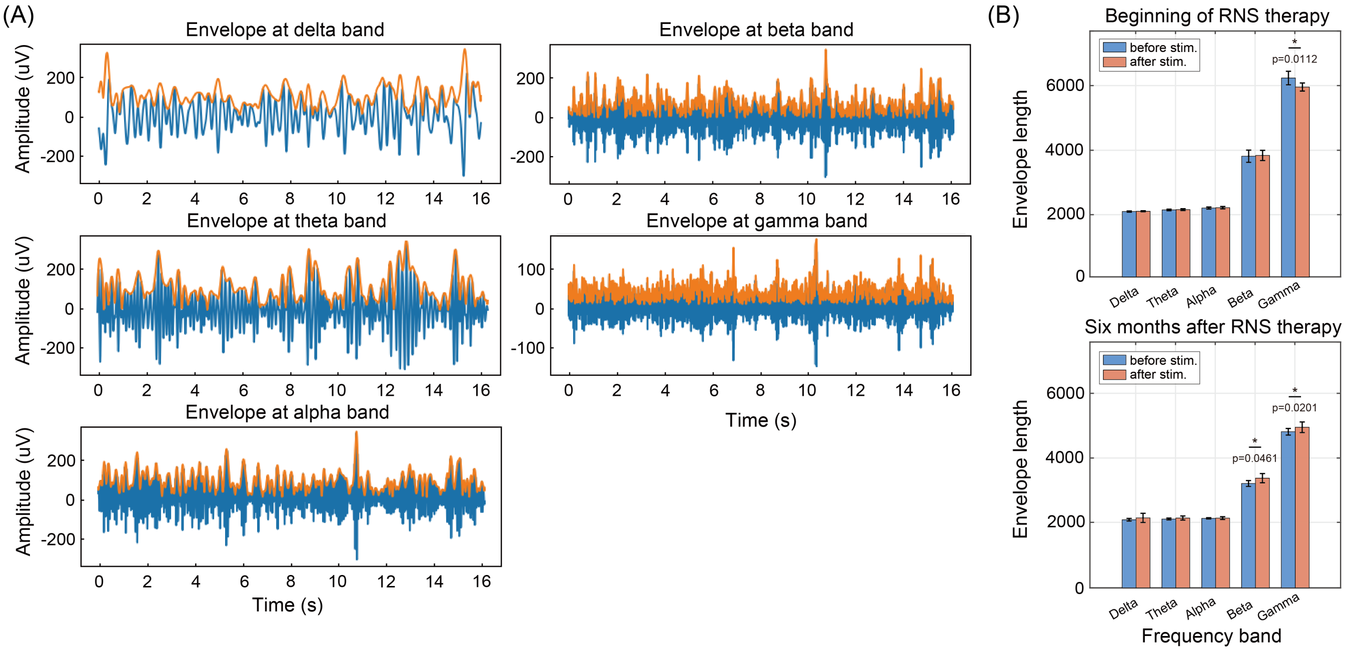

Figure S4: **Scientific utility (envelope dynamics):** envelope length analysis for participant **sub-07** (ses-01 vs. ses-26, ∼six months). (**A**) Hilbert envelopes of band-pass-filtered iEEG signals for Delta, Theta, Alpha, Beta, and Gamma bands; blue and orange traces represent the signal envelopes before and after stimulation, respectively. (**B**) Envelope length (mean ± SD across recordings) by frequency band at therapy initiation (top) and after six months (bottom), comparing pre- (blue) and post-stimulation (orange) windows.

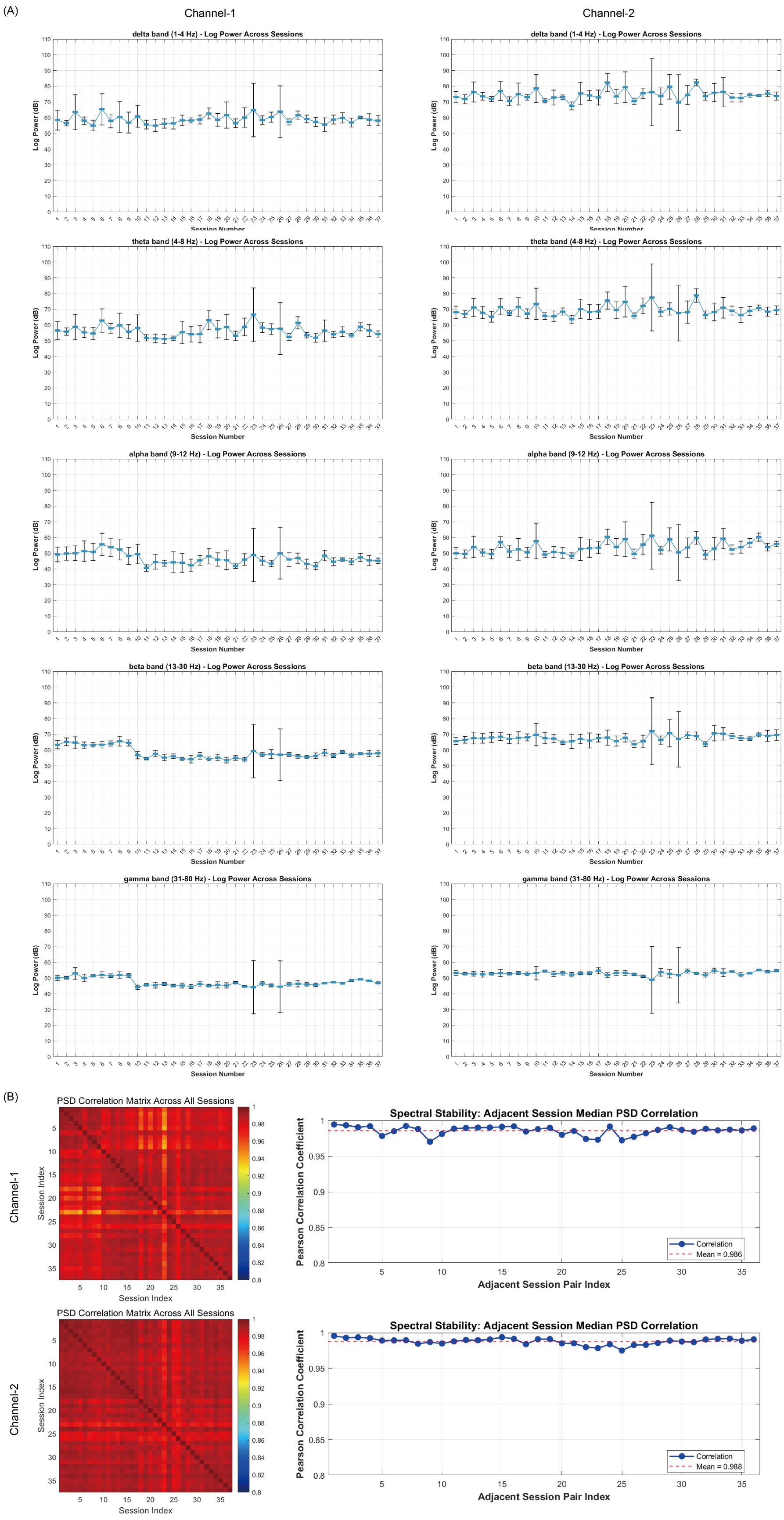

Figure S5: Data quality validation for participant sub-02, following the same pipeline as Figure 2 in the main text.

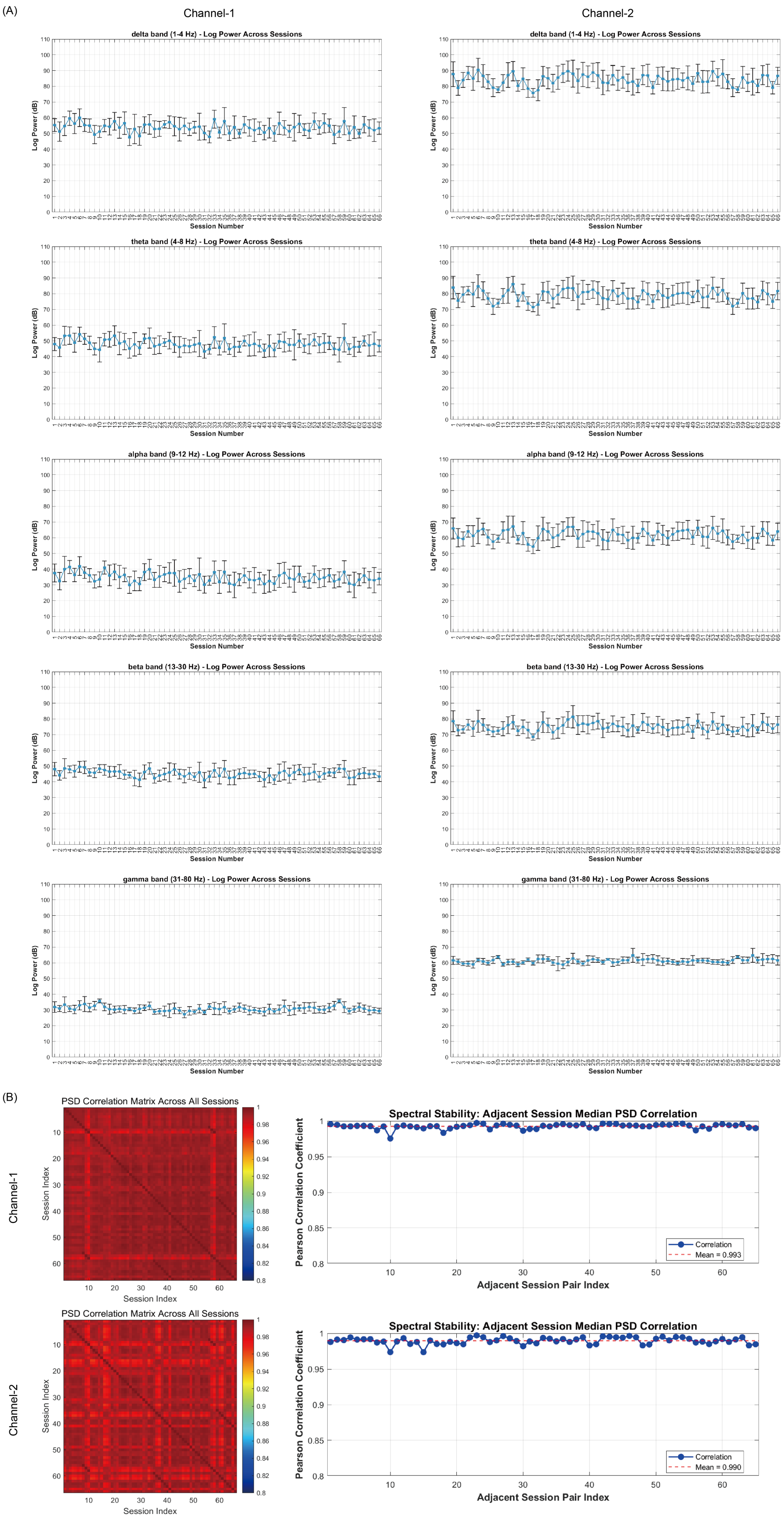

Figure S6: Data quality validation for participant sub-03, following the same pipeline as Figure 2 in the main text.

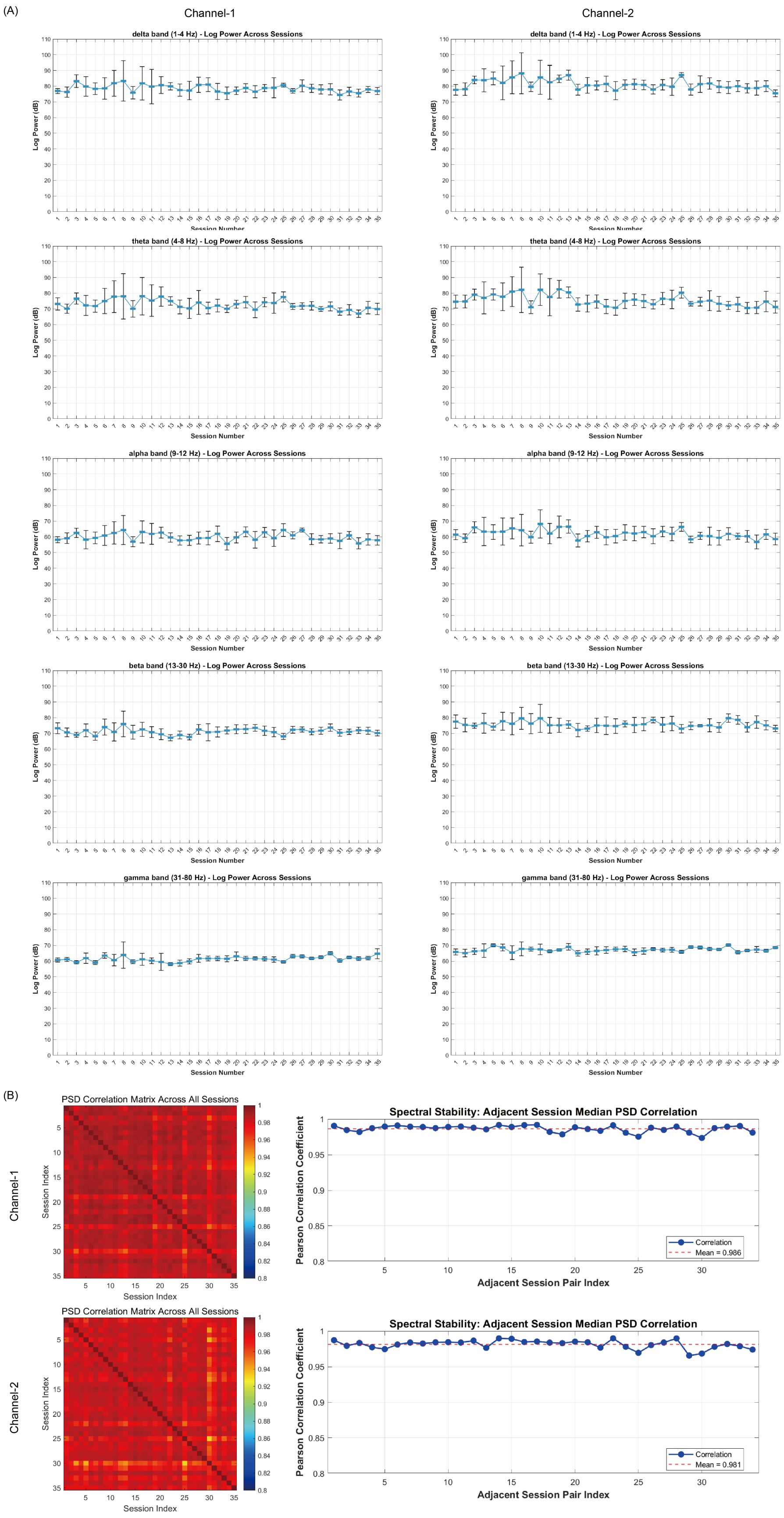

Figure S7: Data quality validation for participant sub-04, following the same pipeline as Figure 2 in the main text.

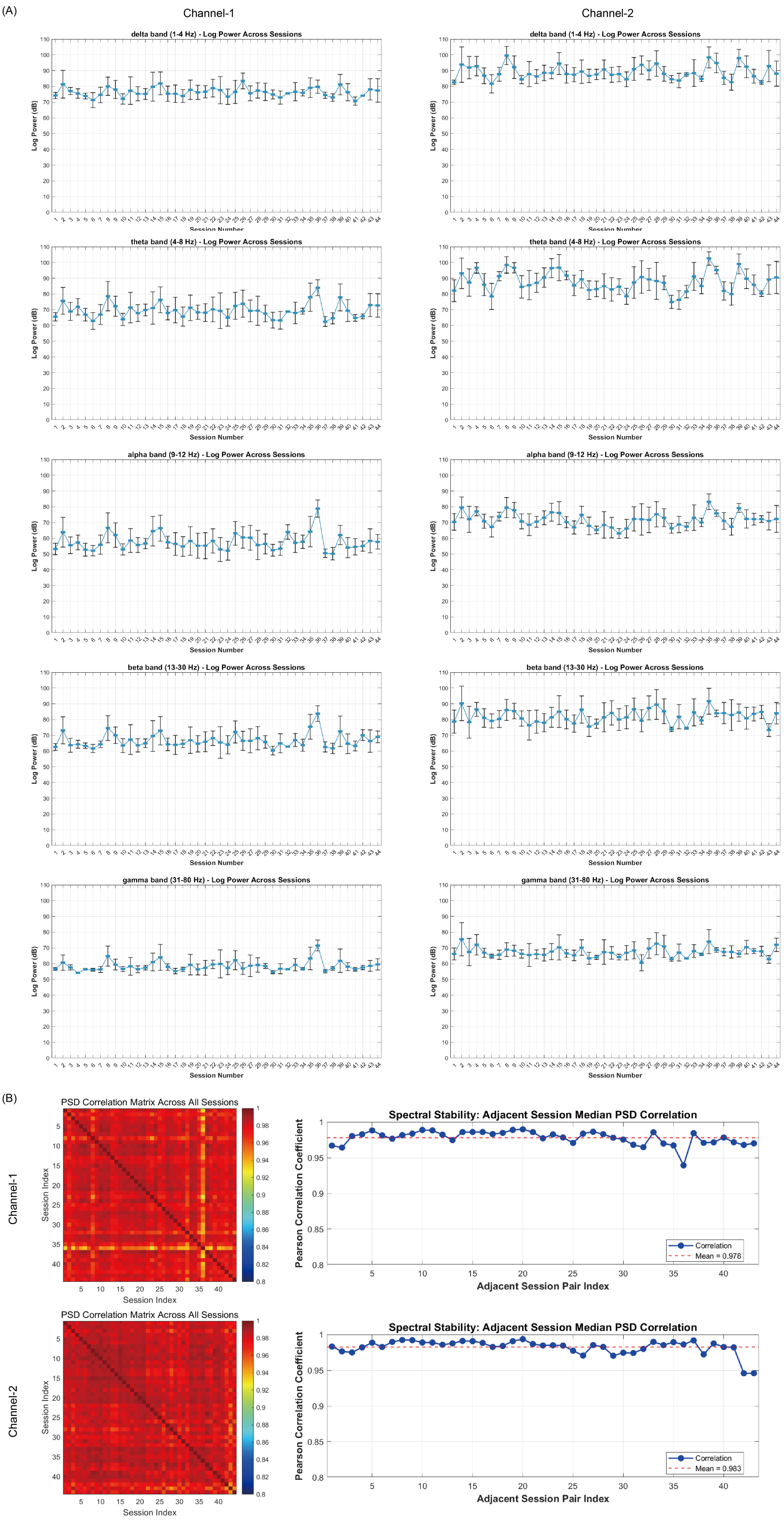

Figure S8: Data quality validation for participant sub-05, following the same pipeline as Figure 2 in the main text.

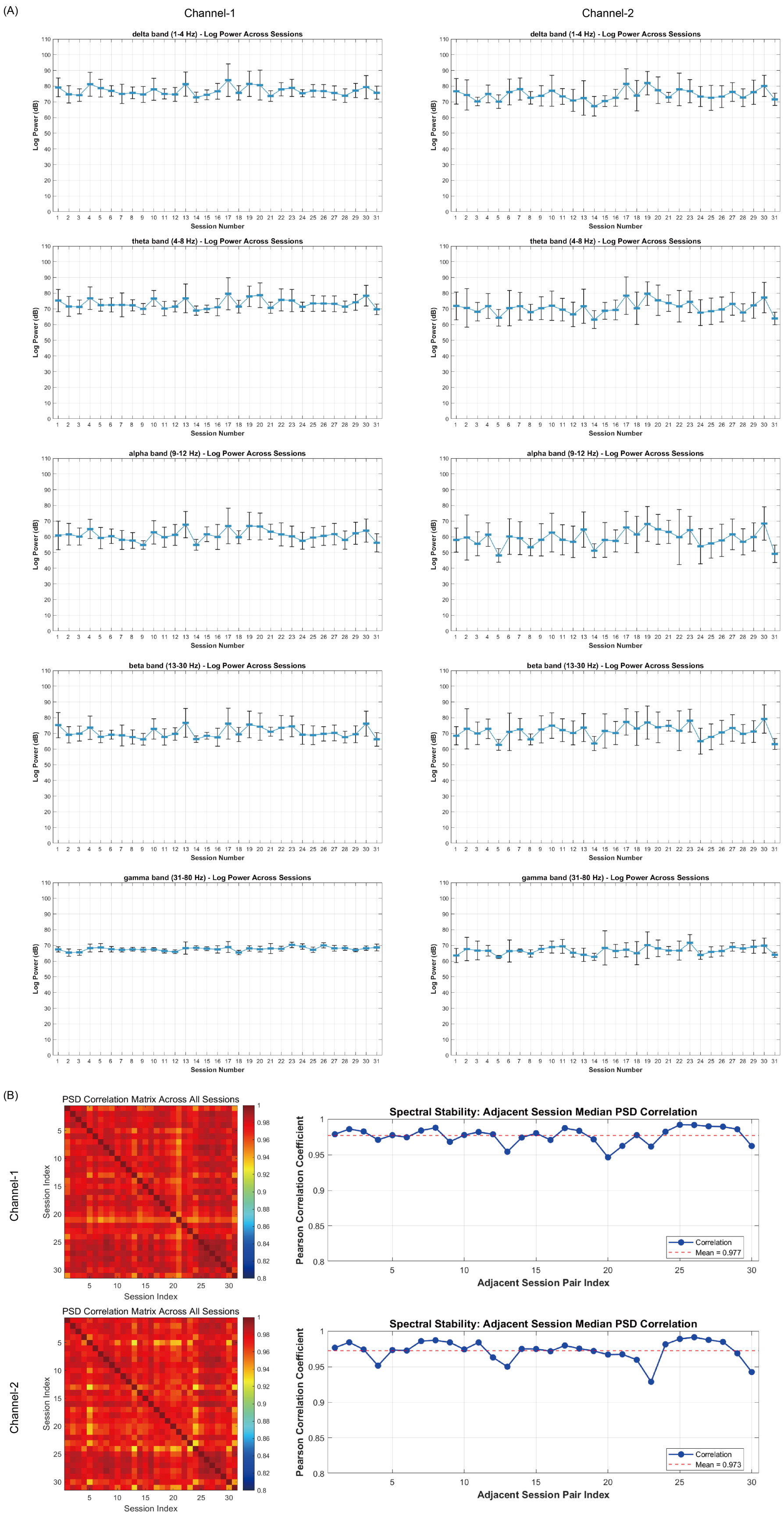

Figure S9: Data quality validation for participant sub-06, following the same pipeline as Figure 2 in the main text.

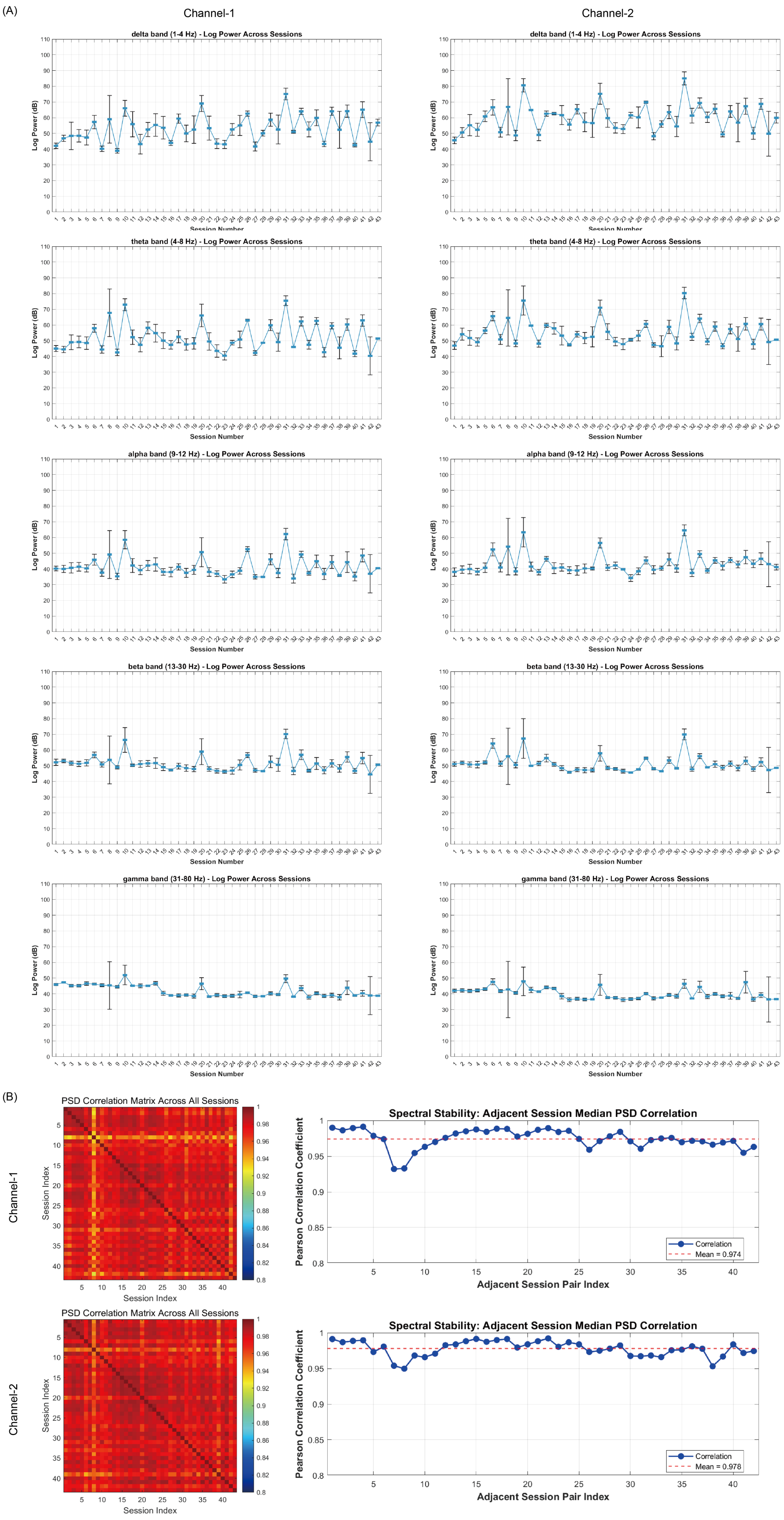

Figure S10: Data quality validation for participant sub-07, following the same pipeline as Figure 2 in the main text.

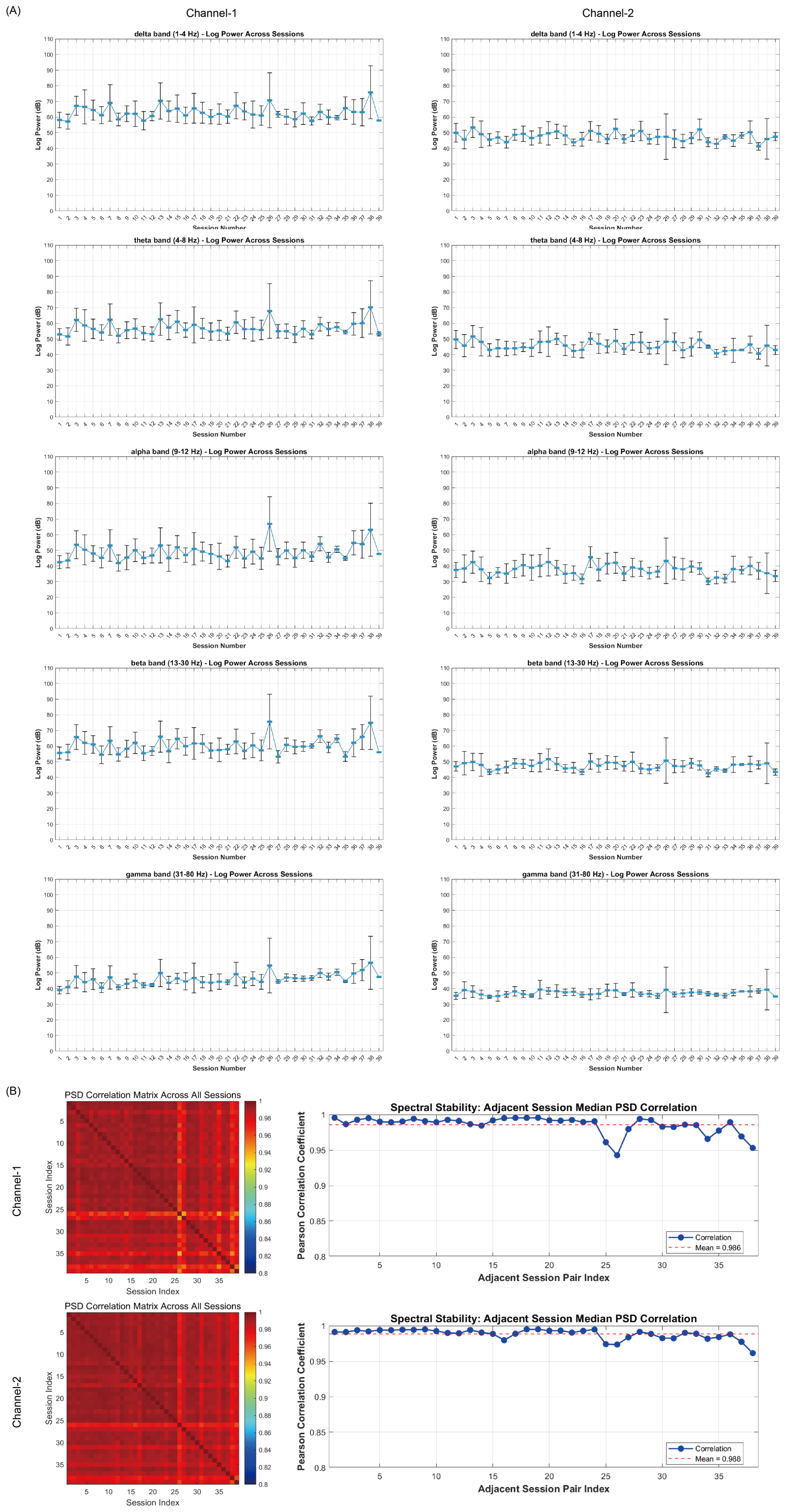

Figure S11: Data quality validation for participant sub-08, following the same pipeline as Figure 2 in the main text.
